# Pretreatment of artesunate promoted hepatocyte proliferation by activating the PI3K/Akt/mTOR signaling pathway in mice

**DOI:** 10.1590/acb394324

**Published:** 2024-10-25

**Authors:** Changyou Lu, Xinkai Li, Chao Fang, Chuntao Li, Yunke Xu, Yong Guo

**Affiliations:** 1 The Affliated Traditional Chinese Medicine Hospital, Southwest Medical University – Department of Hepatobiliary and Pancreatic Surgery – Luzhou (Sichuan) – China.

**Keywords:** Artesunate, Cell Proliferation, Liver Regeneration, TOR Serine-Threonine Kinases

## Abstract

**Purpose:**

Artesunate (ART) has been implicated in regulating the many processes of liver injury, but its roles in liver regeneration still need to be illustrated.

**Methods:**

In the present study, ART was used to pretreat hepatocyte cell line NCTC1469 to study the effect of ART on hepatocyte proliferation *in vitro*. Furthermore, the potency of ART as a regimen to promote liver regeneration and restore liver function was evaluated following partial hepatectomy (PH) on C57BL/6 mice.

**Results:**

ART concentration-dependently promoted hepatocyte proliferation and reduced apoptosis. Cell cycle and Ki-67 immunohistochemical analyses demonstrated that ART supplementation promoted the proliferation of hepatocytes and accelerated liver regeneration. Our results provided evidence that ART improved liver function in a dose-dependent manner, as indicated by decreased serum alanine aminotransferase, aspartate aminotransferase, and increased albumin, and hepatocyte growth factor levels in PH mice. Meanwhile, ART promoted the PI3K/Akt/mTOR signaling in NCTC1469 cells and liver tissue of PH mice. In addition, PI3K inhibitor LY294002 blocked the promotion effect of ART on hepatocyte proliferation and cell cycle progression.

**Conclusion:**

ART promoted hepatocyte proliferation via activation of the PI3K/Akt/mTOR pathway, which was beneficial to liver regeneration of PH-induced liver injury.

## Introduction

The liver is the sole organ in the mammalian body that possesses remarkable regenerative capacity following injury^1^. Following injuries caused by physical, chemical, or biological hazards, the remaining normal hepatocytes can compensate for lost tissue, restore their original mass, and reinstate liver function through cell cycle entry and promotion of cell proliferation. This phenomenon is referred to as liver regeneration (LR), a complex and dynamic process involving multiple pathways and factors^2^^,^ 3.

The capacity of LR is not only essential for the liver to fulfill its function in metabolic homeostasis, detoxification, and storage of nutrients, but also in surgery when partial hepatectomy (PH) and liver transplantation are performed^2^. Various liver diseases, including fibrosis, hepatitis, fatty liver, and failure, frequently result in a significant decline in hepatic regenerative potential and profoundly impact patient prognosis^4^^,^ 5. Especially in patients with cirrhosis, the ability of LR is significantly reduced, which increases the incidence and mortality of liver failure after PH^6^. Therefore, improving LR ability is the basis for the favorable treatment outcome of patients after PH, which can serve as a potential indicator for postoperative survival.

Traditional Chinese medicine (TCM) exhibits distinct advantages in the field of anti-liver fibrosis and anti-liver cirrhosis^7^^,^ 8. Previous studies have predominantly highlighted the hepatocyte proliferative and regenerative properties associated with TCM^9^. It was reported that Baicalin facilitated liver regeneration following alleviating acetaminophen-induced acute liver injury in mice by inducing Nrf2 accumulation in the cytoplasm to promote hepatocyte proliferation^10^. *Codonopsis pilosula*, *Salvia miltiorrhiza* Bunge, *Bupleurum kasi*, and Elephantopus scaber L. promoted liver regeneration and inhibited liver fibrosis in PH rats^11^. In addition, Jie-Du-Hua-Yu granules have been found to effectively prevent acute liver failure induced by D-galactosamine/lipopolysaccharides in rats. This is achieved by stimulating liver regeneration through increased DNA replication and a more favorable cholesterol metabolic ratio^12^.

Artemisinin is a kind of endoperoxide sesquiterpene lactone, which is the only natural source isolated from the TCM Artemisia annul to treat malaria^13^. Artesunate (ART) is a reduced artemisinin succinic acid monoester and an artemisinin derivative with a sesquiterpene lactone structure, which has the best water solubility among all derivatives of artemisinin^14^.

The therapeutic potential of ART in the management of various cancers and other diseases has been widely recognized^15^^,^ 16. The antiproliferative effects of ART have been observed in multiple cancer cell types, leading to cell cycle arrest and subsequent induction of apoptosis, effectively inhibiting tumor progression^17^. The administration of ART effectively attenuated cigarette smoke-induced airway inflammation and oxidative stress, while also significantly inhibited airway smooth muscle cell proliferation through the decreased of α-smooth muscle actin and cyclin D1 expression^18^. It is noteworthy that the combination of ART and sorafenib exhibited potent synergistic anticancer effects against hepatocellular carcinoma cell lines *in vitro*, as well as in the Huh7 cell xenograft model in Balb/c nude mice^19^. However, the effect and molecular mechanism of ART on hepatocyte proliferation and liver regeneration have not been studied.

The extensive establishment of the role of the phosphatidylinositol-3-kinase (PI3K)/protein kinase B (AKT) signaling pathway in the regulation of cell proliferation is well-documented^20^. Upon phosphorylation, AKT exerts its effects on downstream targets, thereby playing a critical role in the regulation of cell cycle and growth^21^. ART has been found to impede the proliferation and migration of retinal pigment epithelial cells, as well as inhibit the epithelial-mesenchymal transition mediated by transforming growth factor-beta 2 (TGF-β2), through the suppression of the PI3K/AKT pathway^22^.

Additionally, the combination of ART and metformin has been shown to effectively mitigate salivary gland damage in rats with type-2 diabetes *mellitus* 23. This protective effect was achieved through the regulation of the PI3K/Akt pathway, resulting in the inhibition of apoptosis and autophagy in the salivary gland^23^. Furthermore, ART has been observed to hinder apoptosis and promote survival in Schwann cells by activating the PI3K/AKT/mTOR axis in cases of diabetic peripheral neuropathy^24^. Nevertheless, there is a lack of clarity regarding the activation of the PI3K/AKT/mTOR signaling pathway in hepatocytes through treatment with ART.

LR has been well studied in the rodent model induced by 2/3 PH^25^. In this study, we investigated the potential role of ART during LR following 2/3 PH in mice. Furthermore, we assessed the effect of ART on hepatocyte proliferation, and further elucidated its underlying mechanism.

## Methods

### Cell treatment

The NCTC1469 murine liver cell line was purchased from Procell (Wuhan, China). The NCTC1469 cells were cultured in a growth medium consisting of DMEM (Gibco BRL, United States of America) supplemented with 10% fetal bovine serum (FBS, Gibco BR L, United States of America) and 1% penicillin-streptomycin solution and maintained in a humidified incubator at 37°C with 5% CO_2_. Then, the cells were treated with ART of 0, 20, 50, and 100 μmol, respectively, and cultured for 24 h. For some experiments, the cells were also pre-treated with the 50 μmol PI3K inhibitor LY294002 (Sigma-Aldrich, St. Louis, MO, United States of America) for 12 h.

### Cell proliferation assay

NCTC1469 cells were enzymatically dissociated and subsequently distributed into 96-well plates at the density of 1 × 10^9^ cells per well. Following this, 100 μL of DMEM medium supplemented with 10% FBS and 10 μL of cell counting kit-8 (CCK-8) solution were introduced into each well, and the cells were incubated for 0.5-2 h within a controlled cell incubator. Subsequently, the absorbance of the samples was quantified at the wavelength of 450 nm.

### Cell apoptosis and cell cycle analysis

NCTC1469 cells were subjected to phosphate-buffered saline (Invitrogen, Carlsbad, CA, United States of America) wash and subsequently diluted to the concentration of 1 × 10^6^ cells/mL. The cells were then suspended in a 150-μL buffer solution. Following this, a staining procedure was conducted at 4°C in darkness for 20 min, utilizing 10 μg/mL Annexin V-FITC and 5 μL PI. Apoptotic cells were analyzed using the BD FACSCelestaTM Flow Cytometer (Becton, Dickinson, and Company). The cell cycle of NCTC1469 cells was assessed using propidium iodide (Beyotime, Shanghai, China; C1052) with the BD FACSCalibur Flow Cytometry System.

### Animals and groups

Male C57/BL6 mice were procured from Chengdu Rongsheng Pharmaceutical Co. [Chengdu, Sichuan; SYXK (Sichuan) 2023-0265]. The mice were provided *ad libitum* access to food and water under controlled environmental conditions, including a temperature range of 20-25°C, relative humidity of 50 ± 1%, and a 12-hour light/dark cycle. The experimental protocol underwent thorough evaluation and received approval from the Experimental Animal Care and Ethics Committee of the Southwest Medical University (No.: 20231023-019). All animal studies adhered to the Animal Research: Reporting of in Vivo Experiments (ARRIVE) guidelines.

**Figure 1:**
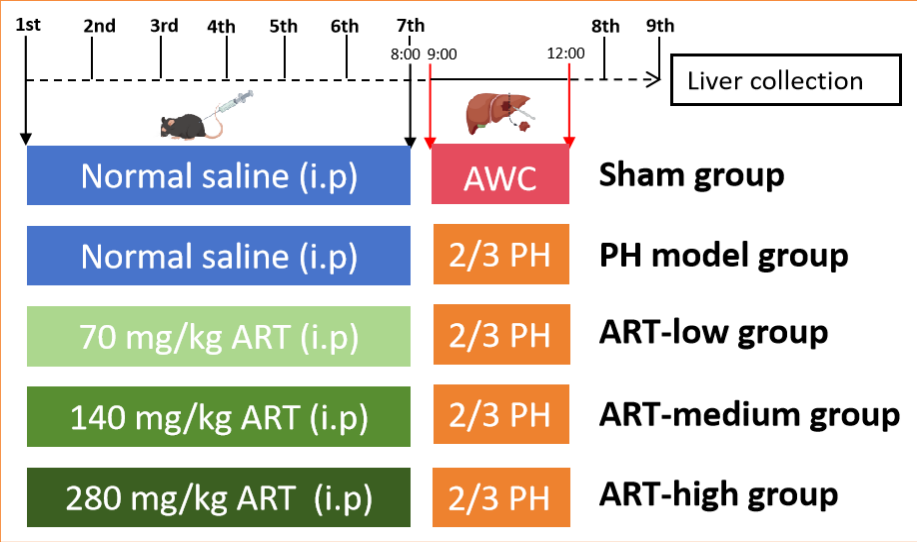
Schematic diagram of animal experiment scheme. Experimental scheme of ART therapy for 2/3 partial hepatectomy mouse model.

PH: partial hepatectomy; ART: artesunate; AWC: abdominal wall closure. Source: Elaborated by the authors

The animal experiment flowchart is shown in Fig. 1. Mice were randomly divided into five groups: sham group, 2/3 PH group, ART low concentration group (ART-low), ART medium concentration group (ART- medium), and ART high concentration group (ART-high). Each group had six mice. ART group mice were intraperitoneally injected with artesunate 70 mg/kg (ART-low), 140 mg/kg (ART-medium), and 280 mg/kg (ART-high) once a day for seven days. Mice in the sham group and PH group were intraperitoneally injected with the same amount of normal saline. Mice in 2/3 PH group performed PH using standard procedures^26^. The 2/3 PH was performed 1 h after intraperitoneal injection of ART at 8 a.m. on day 7.

In short, the surgeries were performed between 9 a.m. and 12 p.m. The mice were anesthetized with 5% isoflurane, and general anesthesia was maintained using 1.5% isoflurane. The lower abdomen of mice was sterilized, and the epidermis and muscle layers were incised a second time to fully expose the liver. Subsequently, the running ligaments and connective membrane between the left lateral lobe and caudate lobe were severed, followed by ligation and resection of the left lateral lobe and middle lobe of the liver. Finally, closure of the incision was performed. In the sham group, only abdominal wall closure was performed. Blood samples were collected 12 h after operation for biochemical analysis, and 48 hours after operation for follow-up experiments.

### Biochemical analysis

The serum levels of aspartate aminotransferase (AST), alanine aminotransferase (ALT), albumin (ALB), and hepatocyte growth factor (HGF) were measured using an automatic biochemical analyzer (Hitachi 7600_020 biochemical automatic analyzer, Tokyo, Japan).

### Western blot analysis

The RIPA buffer (Santa Cruz Biotechnology, Dallas, TX, United States of America) was used to create a lysis solution for liver tissues and NCTC 1469 cells to collect total proteins. The concentration of proteins was determined using the bicinchoninic acid (BCA) protein assay kit (Pierce, Rockford, IL, United States of America). For SDS-PAGE analysis, 30 µg of total cellular protein was loaded and transferred onto nitrocellulose membranes by electrophoresis. Primary antibodies were applied to the filters followed by an HRP-conjugated anti-rabbit-IgG secondary antibody (S0001; Affinity; 1:5,000). The ECL system (Amersham, Piscataway, NJ, United States of America) was utilized for signal development. Densitometric analysis of the results was conducted using Scion Image data analysis software (Scion Corporation, Frederick, MD, United States of America). Table 1 brings information on the corresponding primary antibodies used in this study.

**Table 1:** Antibodies in Western blot.

Primary antibody	Commercial source	Catalog No.	Dilution ratio
CyclinD1	Abclonal, Wuhan, China	A19038	1:2,000
CyclinE	Bioss, Beijing, China	bs-0573R	1:2,000
Phosphorylated-PI3K (p-PI3K)	Affinity, Nanjing, China	AF3241	1:2,000
Phosphorylated-mTOR (p-mTOR)	Cell Signaling Technology, Danvers, MA, United States of America	5536	1:1,000
PI3K	Affinity, Nanjing, China	AF6241	1:2,000
Phosphorylated-AKT (p-AKT)	Abclonal, Wuhan, China	AP0637	1:2,000
mTOR	Abclonal, Wuhan, China	A11355	1:500
AKT	Abclonal, Wuhan, China	A17909	1:2,000
β-actin	Abclonal, Wuhan, China	AC026	1:5,000

Source: Elaborated by the authors.

### Immunohistochemistry stain

Liver tissue sections that were 4-μm thick underwent routine dewaxing and hydration using gradient ethanol. The sections were subsequently subjected to antigen repair solution at temperatures ranging from 95 to 99 °C for 40 min, followed by cooling at room temperature for 20 min. After being washed three times, the sections were treated with a Ki-67 primary antibody (No. ab15580, Abcam, Cambridge, MA, United States of America; 1:200) and incubated overnight at 4°C. Subsequently, goat anti-mouse secondary antibodies labeled with HRP (No. 91196, Cell Signaling Technology, Danvers, MA, United States of America; 1:200) were sliced and incubated at 37°C for 30 min. The EnVision detection and color development kit was then employed for DAB color development, hematoxylin re-staining, gradient ethanol dehydration, xylene transparency, and finally treacle sealing for observation. The resulting immunohistochemistry images were subsequently evaluated.

### TUNEL staining

The paraffin section of liver tissues was dewaxed with different concentrations (100, 95, 80, and 70%) of ethanol. The sections were then exposed to sodium citrate solution for antigenic repair. Deparaffinized brain sections were permeabilized with 0.1% Triton X-100 (ST795, Beyotime, Shanghai, China) for 8 min and incubated with the TUNEL reaction mix at 37°C for 60 min. The sections were re-incubated with 4’,6’-diamidino-2-phenylindole (DAPI, Vector Laboratories, Burlingame, CA, United States of America) before the visualization of the sections with an optical microscope. Green TUNEL dots were identified by the BX53 fluorescence microscope (Olympus, Tokyo, Japan).

### Statistical analysis

A mean and standard deviation is presented for the results. Experimental data were analyzed using Statistical Package for the Social Sciences 22.0 software (IBM Corp., Armonk, NY, United States of America). The data were analyzed by the method of one-way analysis of variance and compared between groups by the method of least variance. The level of statistical significance was established at *p* < 0.05.

## Results

ART promoted hepatocyte proliferation, accelerated the hepatocyte cycle, and reduced apoptosis.

**Figure 2:**
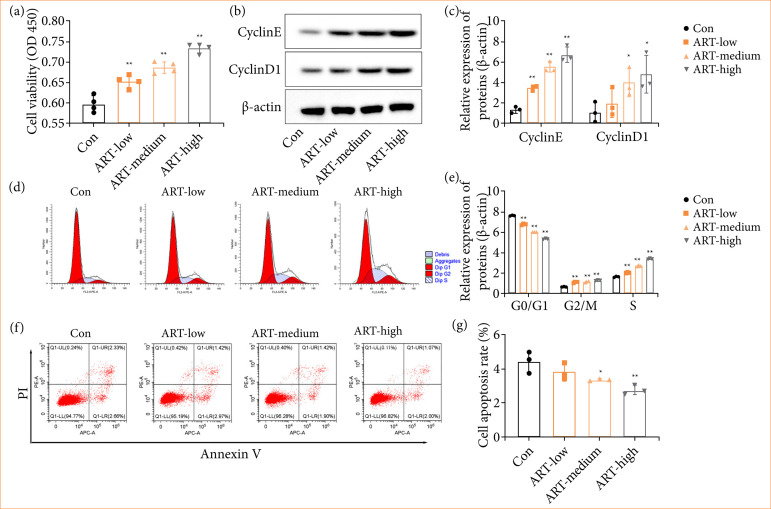
ART promoted hepatocyte proliferation, accelerated hepatocyte cycle, and reduced apoptosis. NCTC 1469 cells were treated with ART of 0, 20, 50 and 100 μmol, respectively, and cultured for 24 h. **(a)** The growth of NCTC 1469 cells was assessed using the CCK-8 assay. (**b** and **c**) Western blot analysis was performed to examine the expression levels of Cyclin D1 and Cyclin E in NCTC 1469 cells. (**d** and **e**) Flow cytometry was utilized to observe the cell cycle progression of NCTC 1469 cells. (**f** and **g**) Flow cytometry was employed to evaluate cell apoptosis in NCTC 1469 cells. Values are means ± standard deviation.

First, we examined the effects of different concentrations of ART on the proliferation and apoptosis of NCTC1469 cells *in vitro*. As shown in Fig. 2a, ART concentration-dependent accelerated the proliferation level of NCTC1469 cells. In addition, the expression of cyclin-related proteins (Cyclin D1 and Cyclin E) in NCTC1469 cells in each group was determined by Western blot (Figs. 2b and 2c). The results showed that ART induced Cyclin D1 and Cyclin E accumulation in a concentration-dependent manner (Figs. 2b and 2c ). We also performed cell-cycle analysis and observed a significant decrease in the G1-phase and an increase in the S-phase, which suggested that ART treatment accelerated cell cycle progression (Figs. 2d and 2e). In addition, flow cytometry data revealed that ART concentration-dependently inhibited apoptosis of NCTC1469 cells (Fig. 2f and 2g).

ART: artesunate; *p < 0.05, **p < 0.01 vs. control group; n = 3/4. Source: Elaborated by the authors.

### ART promoted the PI3K/Akt/mTOR signaling in NCTC1469 cells

**Figure 3:**
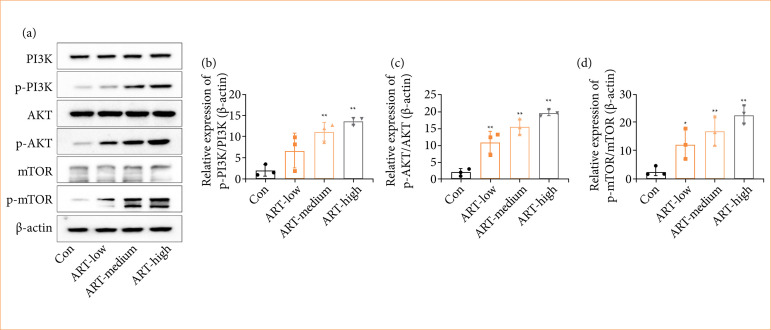
ART promoted the PI3K/Akt/mTOR signaling in NCTC1469 cells. NCTC 1469 cells were treated with ART of 0, 20, 50, and 100 μmol, respectively, and cultured for 24 h. (**a–d**) Western blot was employed to examine the components of the PI3K/AKT/mTOR signaling pathway, encompassing PI3K, phosphorylated-PI3K (p-PI3K), AKT, phosphorylated-AKT (p-AKT), mTOR, and phosphorylated-mTOR (p-mTOR) in NCTC 1469 cells. β-actin was a loading control. Values are means ± standdard deviation.

Mechanistically, the study aimed to investigate the impact of ART on the PI3K/Akt/mTOR signaling pathway. Western blot analysis was utilized to assess the components of this pathway, including PI3K, p-PI3K, AKT, p-AKT, mTOR, and p-mTOR (Figs. 3a–3d). The results demonstrated that ART concentration-dependently enhanced the expression of p-PI3K, p-Akt, and p-mTOR (Figs. 3a–3d). Specifically, the 280-mg/kg ART-treated group exhibited significantly higher levels of p-PI3K, p-Akt, and p-mTOR compared to the groups treated with 140 or 70 mg/kg (Figs. 3a–3d). However, no significant differences were observed in the expression levels of PI3K, Akt, and mTOR following ART treatment (Figs. 3a–3d).

ART: artesunate; *p < 0.05; **p < 0.01 vs. control group; n = 3. Source: Elaborated by the authors.

The promotion effect of ART on hepatocyte proliferation involves the activation of the PI3K/Akt/mTOR pathway.

**Figure 4:**
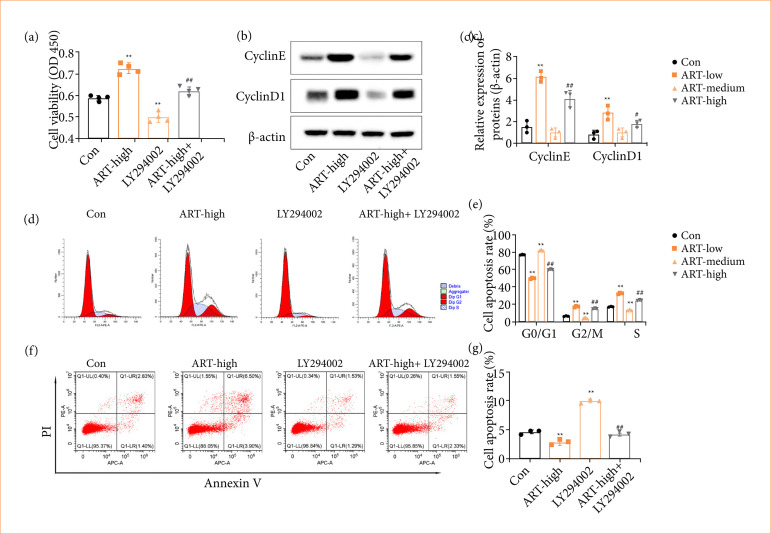
The promotion effect of ART on hepatocyte proliferation involved activation of the PI3K/Akt/mTOR pathway. NCTC 1469 cells were also pre-treated with the 50 μmol PI3K inhibitor LY294002 for 12 h. Meanwhile, NCTC 1469 cells were treated with ART of 0, 20, 50, and 100 μmol, respectively, and cultured for 24 h. **(a)** Cell proliferation in NCTC 1469 cells was quantified by employing the CCK-8 assay. (**b** and **c**) Protein levels of Cyclin D1 and Cyclin E were evaluated in NCTC 1469 cells through Western blot analysis. β-actin was a loading control. (**d** and **e**) Cell cycle progression in NCTC 1469 cells was monitored using flow cytometry. (**f** and **g**) Cell apoptosis in NCTC 1469 cells was assessed via flow cytometry. Values are means ± standard deviation.

CCK-8 assay showed that the proliferative capacity of primary hepatocytes treated with ART was higher than that of untreated hepatocytes. However, p-PI3K inhibitor LY294002 blocked the promotion effect of ART on hepatocyte proliferation (Fig. 4a). Western blot revealed that the expression levels of Cyclin D1 and Cyclin E were increased in ART-treated NCTC1469 cells, which was blocked by LY294002 (Figs. 4b and 4c). Furthermore, when LY294002 was used, cell cycle phase changes stimulated by ART were blocked (Figs. 4c and 4e). As shown in Figs. 3f and 3g, the apoptosis level of NCTC1469 cells was decreased after ART treatment, and, compared with the ART-treated group, the apoptosis level in the ART + LY294002 group markedly increased (Figs. 4h and 4i).

ART: artesunate; *p < 0.05, **p < 0.01 vs. control group; #*p* < 0.05, ##*p* < 0.01 vs. ART-high group; n = 3/4. Source: Elaborated by the authors.

### ART concentration-dependently promoted liver regeneration in vivo

**Figure 5:**
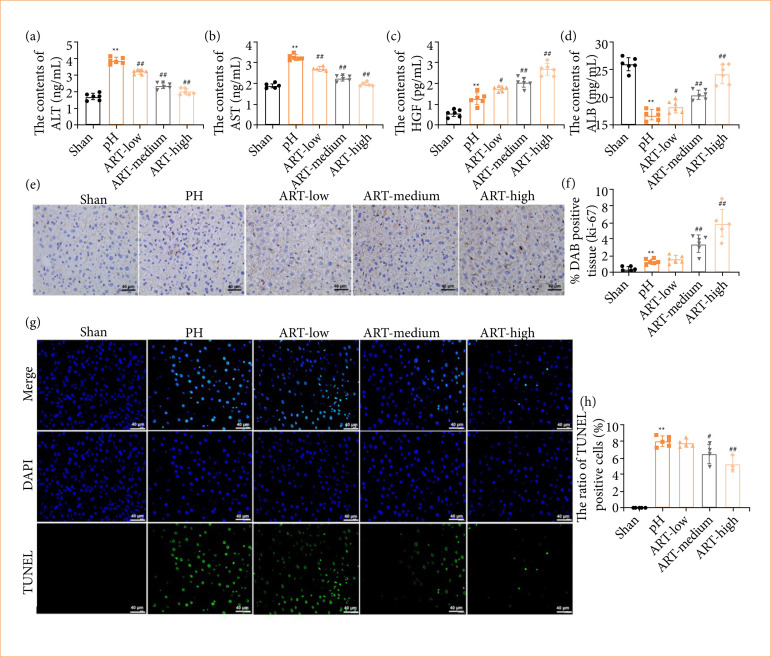
ART concentration-dependently promoted liver regeneration in vivo. Mice were randomly divided into five groups: sham group, 2/3 partial hepatectomy group, ART low concentration group (ART-low), ART medium concentration group (ART-medium), ART high concentration group (ART-high). (**a–d**) The serum levels of alanine transaminase (ALT), aspartate transaminase (AST), total protein, albumin (ALB), and HGF were assayed automatic biochemical detector. (**e** and **f**) Ki67 expression in liver tissues of mice was tested by IHC stain. (**g** and **h**) Cell apoptosis of liver tissues was analyzed using a TUNEL assay. n = 3/4/6. Values are means ± standard deviation.

After ART treatment, the liver function of hepatectomy mice was assessed using an automated biochemical detector. Following PH, the levels of ALT, AST, and HGF in the serum of mice were significantly elevated compared to the sham group, whereas the levels of ALB were significantly reduced (Figs. 5a–5d). The treatment of ART resulted in a significant reduction in ALT and AST levels and increases in ALB and HGF levels in the serum of mice after PH (Figs. 5a–5d). The immunohistochemistry findings confirmed that the expression of the proliferation marker Ki67 was notably promoted in the liver tissue of mice following PH, and this effect was further strengthened after the treatment of medium-dose and high-dose ART (Figs. 5e and 5f). Conversely, the level of apoptosis in the mouse liver tissue exhibited a significant increase after PH, which was concentration-dependently counteracted by ART administration (Figs. 5g and 5h).

ART: artesunate; **p < 0.01 vs. sham; #*p* < 0.05, ##*p* < 0.01 vs. 2/3 partial hepatectomy group. Source: Elaborated by the authors.

### ART concentration-dependently activated the PI3K/Akt/mTOR pathway in liver tissue of PH mice

**Figure 6:**
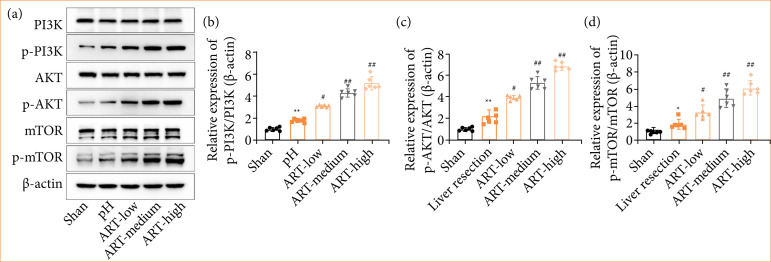
ART concentration-dependently activated the PI3K/Akt/mTOR pathway in liver tissue of PH mice. Mice were randomly divided into five groups: sham group, 2/3 partial hepatectomy group, ART low concentration group (ART-low), ART medium concentration group (ART-medium), ART high concentration group (ART-high). (a-d) The PI3K/AKT/mTOR signaling pathway components, including PI3K, p-PI3K, AKT, p-AKT, mTOR, and p-mTOR, were investigated in liver tissue cells using Western blot analysis. β-actin served as a loading control. Values are means ± standard deviation.

Moreover, the effect of ART on the PI3K/Akt/mTOR pathway was determined by Western blot. Mice in the PH group exhibited significantly increased p-PI3K, p-Akt, and p-mTOR protein expression levels compared with the control group (Figs. 6a–6d). Moreover, the data indicated that protein expressions of p-PI3K, p-Akt, and p-mTOR were further strengthened in ART-treated mice as compared to the untreated group (Figs. 6a–6d). However, the levels of total PI3K, Akt, and mTOR stayed unaltered (Figs. 6a–6d).

ART: artesunate; **p < 0.01 vs. sham; #*p* < 0.05, ##*p* < 0.01 vs. 2/3 partial hepatectomy group. Source: Elaborated by the authors.

## Discussion

ART showcases a wide range of pharmacological effects, encompassing anti-inflammatory properties, immune-modulatory capabilities, and hepatoprotective benefits. The study demonstrated the potential of ART in mitigating liver fibrosis induced by carbon tetrachloride in mice, with its mechanism closely associated with ferritinophagy-mediated hepatic stellate cell ferroptosis^27^. ART increased the sensitivity of sorafenib in anti-hepatocellular carcinoma therapy^19^. In a mouse model of sepsis, ART therapy significantly improved the liver inflammatory response and mitigated the effects of sepsis on liver damage and dysfunction^28^. ART also reduced liver fibrosis caused by schistosomiasis through the downregulation of NDUFB8, a subunit of mitochondrial complex I, and UQCRC2, a subunit of mitochondrial complex III in hepatic stellate cells^29^. Our study further revealed the role of ART in liver regeneration, and it was found that ART significantly stimulated hepatocyte proliferation *in vitro* and liver regeneration in vivo by promoting the expression of the PI3K/AKT/mTOR signaling pathway.

Resting hepatocytes remain in the G0 phase of the cell cycle, maintaining a quiescent state. However, in response to liver tissue loss, hepatic cells exhibit an enhanced capacity for proliferation^30^. Hepatectomy often serves as a trigger for liver regeneration, wherein the remaining hepatocytes re-enter the cell cycle to compensate for the lost tissue^31^.

Studies have demonstrated that LR is a complex biological process, which is intricately regulated by various factors including hormones, growth factors, and neurotransmitters^32^^,^ 33. Following hepatic resection, liver regeneration can be categorized into three distinct phases: initiation (0–6 h), proliferation (12–72 h), and termination (72–168 h)^34^^,^ 35. Hepatocyte proliferation is regulated by several key proteins, including cyclin-dependent kinases (CDKs) that facilitate the transition from G1 to S and G2 to M cell cycles^36^. CDKs can be activated through protein-binding interactions, such as with cyclins^37^^,^ 38. Cyclin D1 and Cyclin E are crucial protein that facilitates cellular entry into the mitotic cycle, and their expression serves as an indicator of tissue cell mitotic activity level while playing a pivotal regulatory role in the stage of cell proliferation^39^. Cyclin D1 is involved in the G1/S phase transition, which is the first stage of the cell cycle, while Cyclin E is involved in the S/M phase transition, which is the second stage^40^^,^ 41.

Notably, the mice exhibited suppressed levels of Cyclin D1 expression and downstream cell cycle proteins following PH^42^. *In-vivo* transfection with Cyclin D1 induced hepatocyte DNA synthesis and the expression of S phase proteins, even in the absence of dietary protein^42^. Furthermore, transfection of hepatocytes with cyclin E promoted hepatocyte proliferation and hyperplasia of the liver^43^. Our *in-vitro* study found that ART promoted hepatocyte proliferation, accelerated hepatocyte cycle, and reduced apoptosis. Meanwhile, ART up-regulated the expression levels of Cyclin D1 and Cyclin E in hepatocytes.

The PI3K/AKT/mTOR pathway is a signaling pathway that plays a crucial role in regulating cell growth, proliferation, and survival^44^. It is involved in various cellular processes, including protein synthesis, metabolism, and cell cycle progression^45^^–^ 47. External stimulation triggers the activation and phosphorylation of PI3K, leading to the binding of p85 to the p110 regulatory subunit for phosphorylating phosphatidylinositol (PI)3 hydroxyl group44. AKT translocates from the cytoplasm to the cell membrane, where it undergoes phosphorylation and activation facilitated by phosphoinositide-dependent kinase (PDK)1 and PDK2^44^. Activated AKT further phosphorylates its downstream target proteins mTOR, Caspase9, NF-κB, FKHR, and so on, thereby mediating subsequent downstream reactions^48^.

The studies have shown that activation of the PI3K/AKT/mTOR pathway promotes hepatocyte proliferation and survival by stimulating protein synthesis, inhibiting apoptosis, and promoting cell cycle progression. The results demonstrate that the PI3K/Akt pathway played a significant role in the process of liver regeneration after hepatectomy in mice^49^. This involvement was mediated by the TGFβ/Smad signaling pathway, facilitated by the interaction between Smad and β-2 Spectrin49. In addition, the osteopontin protein facilitates hepatocyte proliferation both *in vitro* and *in vivo* by activating the PI3K/AKT signaling pathways^50^.

A recent report has indicated that mTOR signaling is crucial for biliary epithelial cell (BEC)-driven liver regeneration in zebrafish^51^, while in mice mammalian target of rapamycin complex 1 (mTORC1) positively regulates liver progenitor cell (LPC) proliferation^52^. Activation of Farnesoid X receptor significantly decreases mTORC1 activation in LPCs during liver regeneration. Notably, inhibition of PI3K or mTORC1 hampers the BEC-driven liver regeneration axis in zebrafish^53^. Moreover, in mice, the activation of the PI3K/AKT/mTOR cell proliferation pathway is facilitated by Panax notoginseng saponins, thereby promoting liver regeneration^54^. Our *in-vivo* study found that ART promoted the liver regeneration of mice after PH surgery by activating PI3K/AKT/mTOR signaling.

## Conclusion

Overall, our study demonstrates that ART could stimulate hepatocyte proliferation by promoting cell cycle progression through the PI3K/AKT/mTOR signaling pathway. ART treatment may be beneficial for liver regeneration following a major hepatectomy. Further animal experimental and clinical studies are required to evaluate its performance and molecular mechanism in promoting liver regeneration in vivo.

## Data Availability

Data will be available upon request.
